# Long-Term Microgravity Exposure Increases ECG Repolarization Instability Manifested by Low-Frequency Oscillations of T-Wave Vector

**DOI:** 10.3389/fphys.2019.01510

**Published:** 2019-12-17

**Authors:** Saúl Palacios, Enrico G. Caiani, Federica Landreani, Juan Pablo Martínez, Esther Pueyo

**Affiliations:** ^1^BSICoS Group, Aragón Institute of Engineering Research, IIS Aragón, Universidad de Zaragoza, Zaragoza, Spain; ^2^Dipartimento di Elettronica, Informazione e Bioingegneria, Politecnico di Milano, Milan, Italy; ^3^CIBER en Bioingeniería, Biomateriales y Nanomedicina, Madrid, Spain

**Keywords:** microgravity, periodic repolarization dynamics (PRD), ventricular repolarization, autonomous nervous system, electrocardiogram (ECG) processing, tilt table test

## Abstract

Ventricular arrhythmias and sudden cardiac death during long-term space missions are a major concern for space agencies. Long-duration spaceflight and its ground-based analog head-down bed rest (HDBR) have been reported to markedly alter autonomic and cardiac functioning, particularly affecting ventricular repolarization of the electrocardiogram (ECG). In this study, novel methods are developed, departing from previously published methodologies, to quantify the index of Periodic Repolarization Dynamics (PRD), an arrhythmic risk marker that characterizes sympathetically-mediated low-frequency oscillations in the T-wave vector. PRD is evaluated in ECGs from 42 volunteers at rest and during an orthostatic tilt table test recorded before and after 60-day –6° HDBR. Our results indicate that tilt test, on top of enhancing sympathetic regulation of heart rate, notably increases PRD, both before and after HDBR, thus supporting previous evidence on PRD being an indicator of sympathetic modulation of ventricular repolarization. Importantly, long-term microgravity exposure is shown to lead to significant increases in PRD, both when evaluated at rest and, even more notably, in response to tilt test. The extent of microgravity-induced changes in PRD has been associated with arrhythmic risk in prior studies. An exercise-based, but not a nutrition-based, countermeasure is able to partially reverse microgravity-induced effects on PRD. In conclusion, long-term exposure to microgravity conditions leads to elevated low-frequency oscillations of ventricular repolarization, which are potentiated following sympathetic stimulation and are related to increased risk for repolarization instabilities and arrhythmias. Tested countermeasures are only partially effective in counteracting microgravity effects.

## 1. Introduction

After almost 60 years of human spaceflight, there is good evidence on detrimental effects on the human body associated with long-term space missions (Williams et al., 2009; Garrett-Bakelman et al., 2019). Two of the main causes underlying those effects are ionizing radiation and changes in gravity conditions. Specifically, microgravity-induced cardiac arrhythmias are a major concern for national space agencies, as very prolonged periods of time in the International Space Station or in a mission to Mars or the Moon might set the stage for the development of ventricular tachycardia or ventricular fibrillation that could end up in sudden cardiac death (Anzai et al., 2014; Caiani et al., 2016). Although the probability of undergoing serious cardiac arrhythmias in the course of a space mission is low, with the estimated probability of suffering a life-threatening event being of 1% per year in short to mid-duration spaceflights (Russomano et al., 2013), currently available data are limited and more sophisticated techniques should be employed to identify potential in-flight abnormalities in the electrical activity of the heart (Convertino, 2009).

Several factors may enhance predisposition to ventricular arrhythmias during spaceflight. Commonly reported bradycardia (Meck et al., 2001), changes in electrolyte composition of blood plasma (Smith and Zwart, 2008), psychological stress (Kanas et al., 2001) and, very relevantly, adaptation of cardiac autonomic modulation (Fritsch-Yelle et al., 1996) may all concur to adversely affect ventricular electrophysiology. In particular, reported alterations in the sympathetic nervous system might contribute to the documented increase in spatio-temporal inhomogeneity of ventricular repolarization, thus potentially providing an electrophysiological substrate for arrhythmias (Caiani et al., 2016). Nevertheless, further evidence on elevated arrhythmic risk during long-term space missions and its underlying mechanisms is yet to be established.

Studies assessing microgravity effects on ventricular repolarization during or immediately after spaceflight are limited. Major findings indicate that long-duration spaceflight prolongs cardiac repolarization, as measured by the QT corrected interval of the electrocardiogram (ECG) (D'Aunno et al., 2003). Due to the limited opportunities to obtain data from humans in space missions, mainly related to the hazards and high costs of spaceflight investigations, several ground-based models have been used to simulate space conditions, explore potential adverse effects associated with weightlessness and assess the effectiveness of proposed countermeasures. Long-term head-down (–6°) bed rest (HDBR) is a ground-based analog widely utilized to simulate microgravity effects on the human body (Pavy-Le Traon et al., 2007; Hargens and Vico, 2016). Relevant alterations in ventricular repolarization have been reported in HDBR studies. In a 90-day HDBR investigation, several subjects were reported to develop QRS-T angles above 100° (Sakowski et al., 2011), with these elevated values having been associated with 3- to 5-fold increased risk for cardiovascular mortality and sudden death in previous works (Kardys et al., 2003; Yamazaki et al., 2005). In another study of only 9- to 16-day HDBR, simulated microgravity was shown to lead to an increase in microvolt T-wave alternans (Grenon et al., 2005), a well-known marker of ventricular arrhythmias and sudden cardiac death. Of note, HDBR-induced increases in T-wave alternans correlated with changes in sympathetic function. In another short-term HDBR study (Mart́ın-Yebra et al., 2015) TWA was, however, shown not to increase during stress-test and tilt-table test after 5- and 21-day HDBR experiments. Interestingly, subjects suffering a more marked orthostatic intolerance after HDBR were found to be those presenting greater values of TWA, even before exposure to simulated microgravity.

A myriad of indices have been reported in the literature to assess ECG repolarization, including prolongation of the QTc interval (Mitchell and Meck, 2004), QT rate adaptation (Pueyo et al., 2004, 2008), QT interval variability (Piccirillo et al., 2009), T-wave alternans (Rosenbaum et al., 1994; Martinez and Olmos, 2005), or T-wave morphological variability (Adam et al., 1984; Badilini et al., 1997; Acar et al., 1999; Baumert et al., 2011; Ramírez et al., 2017a,b), among others. An index of Periodic Repolarization Dynamics (PRD) has been recently proposed to assess sympathetic modulation of ventricular repolarization by measuring low-frequency (below 0.1 Hz) oscillations in the T-wave vector (Rizas et al., 2014). PRD accounts for variability not limited to a specific time interval, as the QT interval or the T-peak-to-T-end interval, but more generally integrating all the spatio-temporal information in the T-wave vector, which can allow for a more robust characterization of beat-to-beat repolarization variations and can provide a better marker to anticipate electrical instabilities (Rizas et al., 2014, 2016).

In this study, ECG signals from healthy volunteers undergoing 60-day HDBR are analyzed. PRD is hypothesized to be able to characterize the effects of sustained simulated microgravity on ventricular repolarization, particularly in response to an orthostatic Tilt-Table Test (TTT), a common procedure used to assess autonomic nervous system function (Zygmunt and Stanczyk, 2010). To overcome identified issues related to angle quantification as part of the PRD technique, a number of updates on the originally reported methods (Rizas et al., 2014, 2016) are also proposed. Additionally, the effectiveness of two different countermeasures, based on exercise and nutrition, to mitigate or reduce microgravity-induced effects on ventricular repolarization during HDBR are assessed.

## 2. Materials and Methods

### 2.1. Study Population

Data from two 60-day –6° HDBR campaigns organized by the European Space Agency (ESA) as part of ESA bed rest studies were analyzed in this work. These studies were conducted between 2015 and 2017 in the :envihab facility of the Institute of Aerospace Medicine at the German Aerospace Center-DLR (Cologne, Germany) and at the Institute of Space Medicine and Physiology-MEDES (Toulouse, France).

For the experiment in Cologne, 22 male volunteers (29 ± 6 years, 181 ± 5 cm, 77 ± 7 kg) were enrolled and randomly distributed into either the countermeasure group (JUMP), who performed 48 training sessions of a varying number of countermovement jumps on a sledge jump system during the HDBR time period (Kramer et al., 2017), or the control group (CTRL), who did not perform any exercise. For the experiment in Toulouse, 20 male volunteers were enrolled (34 ± 7 years, 176 ± 4 cm, 73 ± 7 kg). They were randomly distributed into either the countermeasure group (NUTR), daily receiving a nutritional countermeasure consisting of a cocktail of anti-oxidants and vitamins (daily, 530 mg of polyphenol, 168 mg of vitamin E, 80 μg of Selenium-Solgar®, and 2.1 g of Omega-3—Omacor®), or the control group (CTRL), who did not receive this nutritional integration.

All subjects underwent prior comprehensive medical examination during the selection process and provided written informed consent to participate in the study, which was approved in advance by the respective Ethical Committees for Human Research at the host institutions.

### 2.2. Experimental Protocol

**Figure 1:**
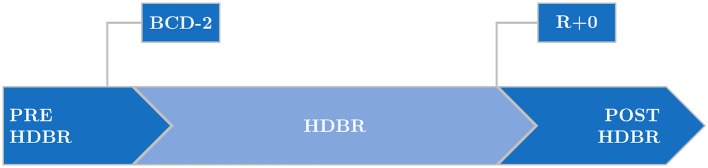
Phases of the head-down bed rest (HDBR) campaign, with indication of the days when volunteers underwent tilt table tests: 2 days before the start of the HDBR period (BCD-2) and just after completing it (R+0).

Both campaigns were divided into three phases: 15 days of PRE-HDBR baseline (BDC-15 to BDC-1), when the subjects became acclimated physiologically and psychologically to the facilities; 60 days of bed rest (HDT1 to HDT60), when subjects were in strict –6° HDBR (24 h/day); and 15 days of POST-HDBR recovery (R+0 to R+14). Figure 1 illustrates these three phases. From HDT1 to HDT60, subjects carried out all activities at –6° HDBR: eating, hygienic procedures (teeth brushing, bowel movement, showering) and free time activities (reading, watching, or using computer). Also, all subjects had the same scheduled wake-up (at 6:30 a.m. and 7:00 a.m. in DLR and MEDES campaign, respectively) and light off (at 11:00 p.m.). More information about the study protocol is available in Kramer et al. (2017).

Two TTTs were performed, one of them 2 days before the start of the HDBR period (BCD-2) and the other one just after completing it (R+0). In each TTT the subject was tilted head-up to an angle of 80° for up to 15 min. If the subject did not experience any presyncopal episode during that time, he was exposed to Lower Body Negative Pressures (LBNP) following a protocol of 3-min –10 mmHg steps for a maximum duration of 15 min. Thirty out of the 84 analyzed recordings did not present presyncopal episodes, of which 18 corresponded to PRE-HDBR and 12 to POST-HDBR.

**Figure 2:**
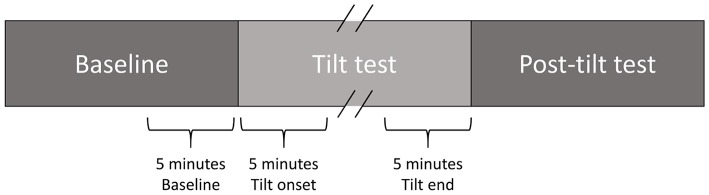
5-min analysis intervals in each TTT.

High-resolution (1,000 Hz) 24-h Holter 12-lead ECG signals (Mortara Instrument) recorded at days BCD-2 and R+0 (both including a TTT) were available for this study. For each TTT, a 5-min interval prior to the start of the tilt phase, the first 5 min immediately following its start and the last 5 min of the tilt phase (possibly including LBNP) were analyzed (Figure 2). If the tilt phase lasted for less than 5 min, its whole duration was analyzed.

### 2.3. ECG Pre-processing

**Figure 3:**
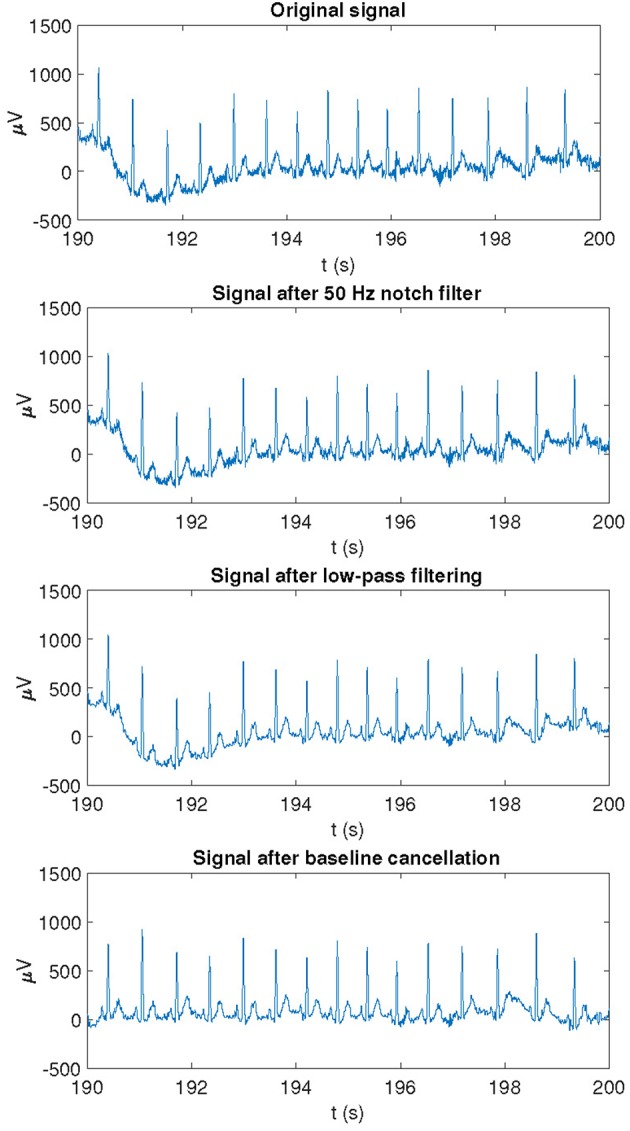
Example of an ECG recording as originally acquired and after application of different pre-processing steps.

Raw ECG signals were pre-processed by a 50 Hz notch filter to remove powerline interference. Taking these pre-processed ECG signals as inputs, QRS detection and ECG wave delineation were performed by using a wavelet-based single-lead automatic system (Martinez et al., 2004). The outputs of the detection and delineation system were combined by using rules to obtain multi-lead ECG delineation marks (Martinez et al., 2004). Since subsequent analysis focused on the T-wave, a 40-Hz low-pass filter was applied to remove noise without altering the T-wave shape. Finally, cubic splines interpolation was applied to estimate and remove baseline wander. An example of an ECG recording as originally acquired and after application of different pre-processing steps is shown in Figure 3.

### 2.4. Calculation of Angles Between Consecutive T Waves

An updated method based on the original method proposed in Rizas et al. (2014) was applied onto the pre-processed ECG signals to compute the angles between consecutive T-waves:

1. Orthogonal leads X, Y, Z were obtained from the 12-lead ECG by using the inverse Dower matrix (Edenbrandt and Pahlm, 1988).
2. The onset and end of each T-wave, denoted by T_*on*_ and T_*off*_, were identified by the delineation system described above. When the delineation failed to identify a T-wave onset (T-wave offset, respectively) for a given beat, its location was set based on T_*on*_ (T_*off*_, respectively) locations for adjacent beats with respect to their corresponding QRS positions. Specifically, T_*on*_ (or T_*off*_) was located at a distance from the corresponding QRS fiducial point equal to the median interval between the QRS and T_*on*_ (or T_*off*_) positions of 30 beats around that beat.
  As T-wave boundaries change on a beat-to-beat basis, and may be influenced by delineation errors, the angle between each two consecutive T-waves was computed by defining a unique temporal window for both waves being analyzed. Specifically, for each angle calculation, the window onset was set at the latest T_*on*_ of both analyzed beats computed with respect to their QRS fiducial points, while the window end was set at the earliest T_*off*_ of both beats computed from their QRS fiducial points.
3. A constant value was subtracted from each T wave in each of the analyzed leads so that the amplitude at T_*off*_ was set to 0 mV. Subsequently, an average T-wave vector was calculated for each T-wave. The angle dT° between two consecutive T-waves, which is associated with the instantaneous degree of repolarization instability, was calculated by using the dot product of each pair of consecutive average T-wave vectors.
4. The dT° time series was filtered by using a 10th-order median filter to attenuate outliers and avoid very abrupt changes in the time series.

### 2.5. PRD Computation

**Figure 4:**
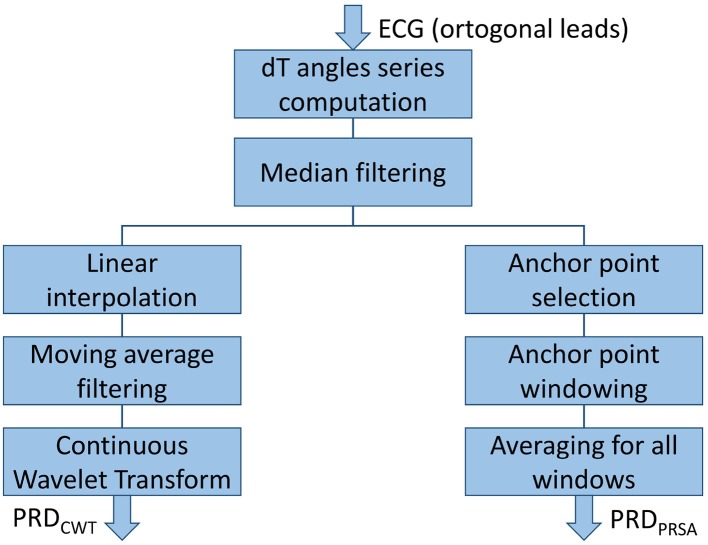
Steps applied to calculate PRD (both CWT- and PRSA-method).

Two different methods, based on Continuous Wavelet Transform (CWT) and Phase-Rectified Signal Averaging (PRSA), respectively, were developed based on the initial methodology proposed in Rizas et al. (2014, 2016). These methods were tested for quantification of the low-frequency components of the beat-to-beat dT° series. The steps followed in each of the two methods are depicted in Figure 4.

#### 2.5.1. PRD Computation Using Continuous Wavelet Transform

CWT is one of the most widely-used tools for time-frequency analysis (Addison, 2005). Based on the dT° series calculated as described in section 2.4, the next steps were followed to compute PRD (Rizas et al., 2014):

5. The dT° series was linearly interpolated at 2 Hz and a 10-sample moving average filter was used to remove artifacts.
6. CWT was computed at all scales from 1 to 40 by using a 4th-order Gaussian wavelet to quantify low-frequency oscillations of dT°. Wavelet coefficients were obtained for each scale at each time point and an average wavelet coefficient was computed for each scale.
7. Scales (*a*) were converted to pseudo-frequencies (*F*_*a*_, expressed in Hz) according to the following equation (Abry, 1997):
  (1) $$\begin{array}{r} F_{a}=\frac{F_{c}}{a\cdot \Delta } \end{array}$$

where *F*_*c*_ is the center frequency of the mother wavelet, in Hz, and Δ denotes the sampling period, in seconds.

PRD_CWT_ was defined as the average wavelet coefficient in the frequency range between 0.025 and 0.1 Hz.

#### 2.5.2. PRD Computation Using Phase-Rectified Signal Averaging

An alternative method to compute oscillatory fluctuations, with less computational requirements, has been proposed based on PRSA (Bauer et al., 2006). The following steps were followed to compute PRD from the dT° series (Rizas et al., 2016):

1. Anchor points were defined by comparing averages of *M* = 9 values of the dT° series previous and posterior to the anchor point candidate (*x*_*i*_). A beat *i* is considered an anchor point if:
  (2) $$\begin{array}{r} \frac{1}{M}\overset{M-1}{\underset{j=0}{\sum }}x_{i+j}>\frac{1}{M}\overset{M}{\underset{j=1}{\sum }}x_{i-j} \end{array}$$
2. Windows of 2 *L* values were defined around each anchor point. If an anchor point was so close to the beginning or to the end of the dT° series that there were not enough samples before or after it, it was disregarded. In this study, *L* = 20 was chosen because it was the minimum value to detect frequencies in the range of interest (0.025–0.1) Hz.
3. PRSA series were obtained by averaging the dT° series over all defined windows.

PRD_PRSA_ was defined as the difference between maximum and minimum values of the PRSA series.

### 2.6. Heart Rate Variability Analysis

RR interval series were computed from the QRS detection marks obtained in section 2.3 for all analyzed 5-min segments at baseline as well as at the beginning and end of TTT. Instantaneous heart rate (HR) variability (HRV) series were calculated following the method described in Bailón et al. (2011). For each segment, the power spectral density (PSD) of HRV was computed by using the periodogram method. A high-frequency band (HF, [0.15, 0.4] Hz) and a low-frequency band (LF, [0.04, 0.15] Hz) were defined for HRV analysis in the frequency domain and the LF and HF powers were calculated by integrating the power spectrum in each of those two bands, respectively. The normalized LF power (LFn), the normalized HF power (HFn) and the ratio between the power in the LF and HF bands (LF/HF) were computed (Malik et al., 1996). Also the median HR (HR_median_) was computed.

### 2.7. Statistical Analysis

The Mann-Whitney *U*-test (or Wilcoxon rank-sum test) was used to compare independent samples, as when comparing each countermeasure (JUMP or NUTR) subgroup vs the corresponding CTRL subgroup. Wilcoxon signed-rank test was used for comparison of paired samples, as when comparing changes induced by HDBR or by TTT in a given group of subjects. Spearman's correlation coefficient ρ and Kendall's τ were used to quantify rank correlation between CWT and PRSA. All statistical analyses were carried out using MATLAB R2017a (9.2).

## 3. Results

### 3.1. Comparison of PRD Computed by CWT- and PRSA-Based Methods

**Figure 5:**
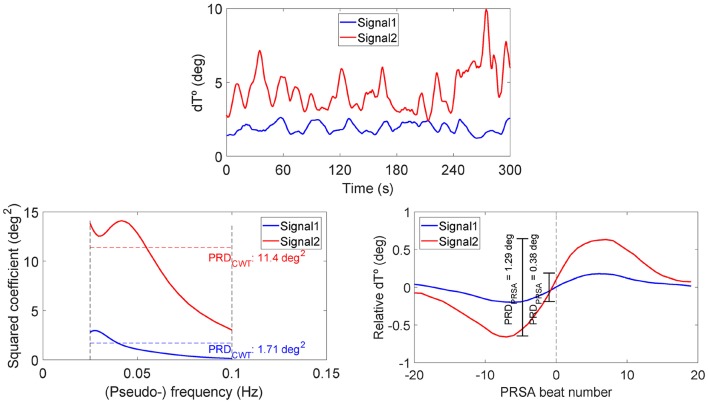
Examples of dT° series (upper), frequency pseudospectra (bottom left), and PRSA series (bottom right) for two ECG segments from a volunteer of the study, at PRE-HDBR and at POST-HDBR, presenting remarkably different magnitudes of low-frequency oscillations in ventricular repolarization. Associated PRD values are indicated in the bottom panels, as computed using CWT- and PRSA-based methods.

Figure 5 shows the two analyzed recordings, at PRE-HDBR and POST-HDBR, from a volunteer presenting small and large magnitudes of low-frequency oscillations in ventricular repolarization, respectively. The three plots represent the dT° series, the frequency pseudospectra (in terms of squared wavelet coefficients) and the PRSA series. The blue line corresponds to the ECG segment at PRE-HDBR and the red one to the ECG segment at POST-HDBR. Note that the case shown in blue presents low-frequency oscillations in dT° of small magnitude, which translates into low values of PRD_CWT_ and PRD_PRSA_. The red case, in contrast, presents low-frequency oscillations in dT° of larger magnitude and is associated with considerably higher PRD values, both when measured by using CWT- and PRSA-based methods.

**Figure 6:**
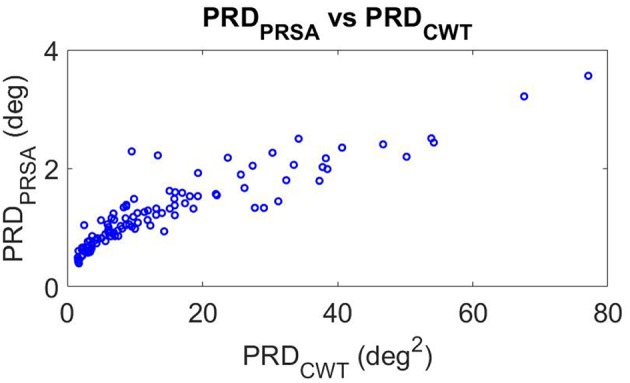
Scatterplot of PRD values measured by CWT- and PRSA-based methods.

Figure 6 shows the correlation of PRD values computed by using the CWT-based method (X-axis) and the PRSA-based method (Y-axis) for all analyzed segments (baseline, beginning, and end of the tilt phase) in the CTRL group of DLR and MEDES campaigns, for both PRE-HDBR and POST-HDBR. The scatterplot shows a strong correlation between both methods. Rank correlation coefficients were: Spearman's ρ = 0.93 (*p* < 10^−50^), Kendall's τ = 0.79 (*p* < 10^−35^). In the following, all presented results use the PRSA-based method.

### 3.2. Tilt Test-Induced Effects on PRD

**Figure 7:**
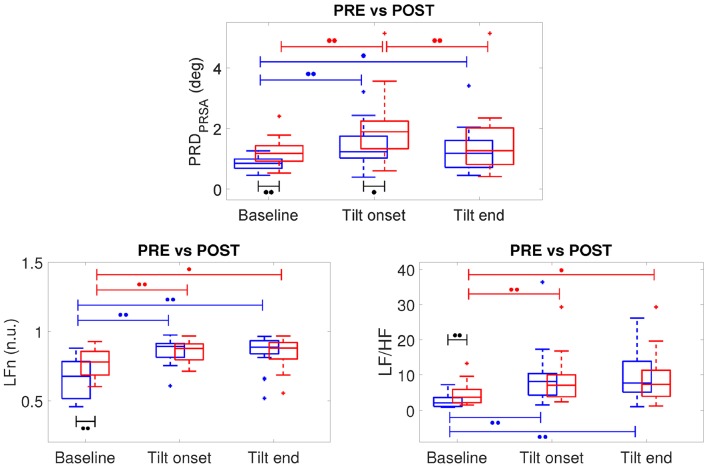
Boxplots of PRD, LFn and LF/HF at PRE-HDBR (in blue) and POST-HDBR (in red) evaluated at baseline and at the beginning and end of the tilt phase. ^∙∙^*p* < 0.01, ^∙^*p* < 0.05 (Wilcoxon signed-rank test).

Figure 7, top panel, shows the results of the analysis of three 5-min segments of the dT° series, corresponding to baseline (prior to the tilt) as well as to beginning and end of the tilt phase, for all volunteers in the CTRL group of both campaigns (DLR and MEDES). Results are separately presented for PRE-HDBR (before HDBR) and POST-HDBR (after HDBR). As can be observed from the figure, PRD increased following tilt as compared to baseline, being the results statistically significant when the segment at the beginning of the tilt phase was analyzed. This was true for both PRE-HDBR and POST-HDBR. Results on the effects of tilt on the HRV indices LFn and LF/HF are presented in the bottom panels of Figure 7. Both indices showed significantly larger values in response to tilt, indicating increased sympathetic drive during orthostatic stress, both at PRE-HDBR and POST-HDBR. The effect of tilt on other HR and HRV indices is presented in Figure S1, which shows a significant increase of HR_median_ and a significant decrease of HFn in response to tilt.

### 3.3. Microgravity-Induced Effects on PRD

The effects of microgravity exposure on PRD obtained by comparing PRE-HDBR and POST-HDBR for CTRL group of DLR and MEDES campaigns can be observed from Figure 7. At baseline (before TTT), PRD was significantly increased at POST-HDBR with respect to PRE-HDBR, changing from 0.85 [0.31] deg at PRE-HDBR to 1.18 [0.51] deg at POST-HDBR (*p* < 0.01), as presented in the left columns of Figure 7.

Considering the analysis at the onset of the tilt phase, PRD was also increased at POST-HDBR with respect to PRE-HDBR, changing from 1.24 [0.72] deg at PRE-HDBR to 1.89 [0.91] deg at POST-HDBR (*p* < 0.05), as shown in the middle columns of Figure 7.

No statistically significant differences in PRD at POST-HDBR vs. PRE-HDBR were observed when analyzed at the end of the tilt phase (right columns in Figure 7), although there was a trend to increased PRD at POST-HDBR as compared to PRE-HDBR. Specifically, PRD increased from 1.18 [0.88] deg at PRE-HDBR to 1.27 [1.21] deg at POST-HDBR (n.s.).

In the case of the HRV indices LFn and LF/HF, statistically significant microgravity-induced increases were observed when the baseline period was analyzed (see Figure 7). Specifically, LFn changed from 0.67 [0.27] n.u. at PRE-HDBR to 0.78 [0.17] n.u. at POST-HDBR (*p* < 0.01). LF/HF changed from 2.12 [2.51] at PRE-HDBR to 3.74 [3.78] at POST-HDBR (*p* < 0.01). The index HFn significantly decreased from PRE-HDBR to POST-HDBR when evaluated at baseline, whereas HR_median_ was significantly augmented due to microgravity either when evaluated at baseline or at the beginning and end of the tilt test (see Figure S1).

### 3.4. PRD and HRV Relation

Figure S2 shows the relationship between tilt-induced changes in PRD and in HR or HRV indexes (LFn, LF/HF and HR_median_) at PRE-HDBR and POST-HDBR. No significant correlation could be found between PRD and HR or HRV, with Spearman's correlation coefficient ρ being below 0.15 in all evaluated cases (n.s.).

### 3.5. Effectiveness of Exercise-Based Countermeasure

**Figure 8:**
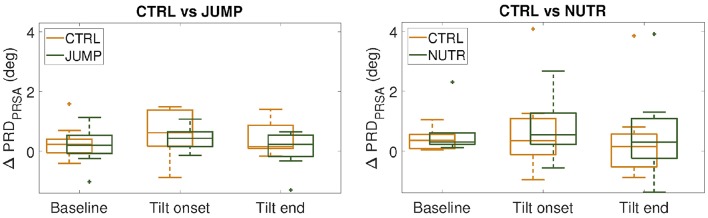
ΔPRD values (median ± IQR) measured respect to PRE-HDBR for the CTRL subgroups (in orange) and the countermeasure (JUMP or NUTR) subgroups (in green) at baseline, beginning and end of the tilt phase. (n.s., Wilcoxon rank-sum test).

The ability of a jump-based countermeasure to reverse the effects of microgravity was evaluated by comparing PRD values at PRE-HDBR and POST-HDBR in each of the CTRL and JUMP subgroups of the DLR campaign. The values for PRD measured for each phase of the TTT are presented in Table 1. Although there were increases in PRD values from PRE-HDBR to POST-HDBR in both CTRL and JUMP subgroups, the increase was much more attenuated in the JUMP subgroup. Significant differences were found at the beginning of tilt for both CTRL and JUMP subgroups. Illustration of the effects of the JUMP countermeasure are presented in Figure 8 (left panel), which shows values of ΔPRD, calculated as the PRD value at POST-HDBR minus the PRD value at PRE-HDBR for each subject. From the figure it is clear that whereas values of ΔPRD were clearly positive in the CTRL subgroup, particularly during the tilt phase, values were remarkably closer to 0 in the JUMP subgroup.

**Table 1:** PRD values (median [IQR]) at all phases of TTT for PRE-HDBR and POST-HDBR in CTRL and JUMP subgroup.

******PRD******		**PRE-HDBR (deg)**	**POST-HDBR (deg)**
Baseline	CTRL	0.78 [0.40]	0.93 [0.79]
	JUMP	0.72 [0.34]	0.81 [0.47]
Tilt onset	CTRL	1.32 [1.40]	2.04 [0.99]^*^
	JUMP	0.77 [0.29]	1.28 [0.77]^*^
Tilt end	CTRL	0.95 [0.98]	1.48 [1.12]
	JUMP	0.85 [0.51]	1.02 [0.59]

* *p < 0.05 (with respect to PRE-HDBR)*.

### 3.6. Effectiveness of Nutrition-Based Countermeasure

Results on the effectiveness of a nutrition-based countermeasure are presented in Table 2. Figure 8 (right panel) illustrates these results in terms of ΔPRD (differences between POST-HDBR and PRE-HDBR calculated for each subject in the analyzed subgroups). As can be observed from Table 2, baseline PRD increased significantly from PRE-HDBR to POST-HDBR in both the CTRL and NUTR subgroups of the MEDES campaign. When evaluation was performed at the beginning of the tilt test, PRD increased at POST-HDBR with respect to PRE-HDBR, although differences were not statistically significant. At the end of the tilt phase, PRD showed a trend of increase in the NUTR group but not in the CTRL group. Results shown in Figure 8 (right panel) confirm the lack of effectiveness of the evaluated nutrition-based countermeasure.

**Table 2:** PRD values (median [IQR]) at all phases of TTT for PRE-HDBR and POST-HDBR in CTRL and NUTR subgroup.

******PRD******		**PRE-HDBR (deg)**	**POST-HDBR (deg)**
Baseline	CTRL	0.92 [0.19]	1.32 [0.33]^**^
	NUTR	1.10 [0.51]	1.22 [1.04]^*^
Tilt onset	CTRL	1.22 [0.23]	1.59 [0.98]
	NUTR	1.32 [1.02]	1.93 [1.72]
Tilt end	CTRL	1.27 [0.88]	1.13 [0.96]
	NUTR	1.19 [0.74]	1.41 [1.76]

** *p < 0.01*,

* *p < 0.05 (with respect to PRE-HDBR)*.

## 4. Discussion

This study aimed at investigating alterations in ventricular repolarization associated with long-term exposure to simulated microgravity conditions elicited by 60-day HDBR. Two methods have been developed for quantification of low-frequency oscillations in the T-wave of the ECG, departing from the original methodology proposed in Rizas et al. (2014, 2016). These methods, one using CWT and the other one using PRSA, have been shown to render concordant results in terms of the index of Periodic Repolarization Dynamics, PRD, a marker of low-frequency repolarization oscillations whose increase has been shown to be predictor of ventricular arrhythmias and sudden cardiac death (Rizas et al., 2014, 2016). This study has proved that microgravity remarkably enhances PRD, particularly when evaluated in response to sympathetic stimulation induced by tilt test. An exercise-based countermeasure has been shown to partially reverse microgravity-induced effects on PRD, whereas a nutrition-based countermeasure has been shown not to be effective at all.

The methods developed in this study for PRD quantification departed from the CWT- and PRSA-based methods proposed in Rizas et al. (2014, 2016), respectively. Whereas, the CWT-based method in Rizas et al. (2014) used spherical coordinates, our method used Cartesian coordinates, which rendered improved results for cases where T-wave vectors were close to the axes. Also, our method introduced a refinement on the temporal window used for T-wave definition so as to guarantee that the two consecutive T-waves involved in each angle computation had comparable T-wave window beginnings and ends with respect to their corresponding QRS fiducial points. An additional difference regards the number of samples used for the moving average filter, which was 30 in Rizas et al. (2014) and 10 in our study to minimize distortion of relevant information in the frequency band of interest. For our updated CWT- and PRSA-based methods, correlation analysis has confirmed a strong agreement between them. Although our PRD values are notably different from those obtained in Rizas et al. (2014, 2016), the agreement between CWT- and PRSA-based methods is in concordance with the findings reported in Rizas et al. (2016), where an approach based on PRSA was presented as an alternative to the approach using the CWT technique. The advantage of the PRSA approach is that it highly reduces the computational cost associated with PRD computation.

The analysis conducted in this work has shown that the autonomic changes induced by TTT are manifested as an increase in PRD, both when measured at PRE-HDBR and at POST-HDBR. Such PRD changes could be attributable to an increased sympathetic drive, as indicated by increases in the HRV indices LFn and LF/HF, in line with many other HRV studies, including the ones pioneering spectral HRV analysis during TTT (Pagani et al., 1986, 1988). It is well-known that sympathetic stimulation influences ventricular repolarization and modifies the characteristics of the T-wave in the ECG (Ramirez et al., 2011). Our results showing an increase in PRD in response to TTT are in line with the changes in PRD reported in response to variations in sympathetic activity or β-adrenergic modulation (Rizas et al., 2014, 2016). In our study, those changes are shown not to be explained by HRV changes but to reflect direct autonomic modulation of the ventricular myocardium, in accordance with the findings reported in Rizas et al. (2014, 2016). *In vivo* studies in patients have demonstrated that the same low-frequency oscillatory behavior of ventricular repolarization occurs locally, as measured from activation recovery intervals (ARIs) obtained from unipolar epicardial electrograms during ventricular pacing (Hanson et al., 2014; Porter et al., 2018). In those studies, heightened arousal of the sympathetic nervous system was elicited and maintained by mental stress or by Valsalva maneuver, which allowed characterization of low-frequency oscillations in ARI, a surrogate of action potential duration (APD), showing that those oscillations are coupled to oscillations in systolic and diastolic blood pressure (Hanson et al., 2014; Porter et al., 2018). Computational studies have provided insight into the mechanisms underlying sympathetically-mediated low-frequency oscillations of APD and the observed inter-individual differences (Pueyo et al., 2016; Sampedro-Puente et al., 2019). Specifically, phasic changes in both β-adrenergic stimulation and hemodynamic loading, a known accompaniment of enhanced sympathetic activity, have been demonstrated to contribute to low-frequency oscillations in APD, with these two actions being synergistic (Pueyo et al., 2016). Ionic differences in the densities of the L-type calcium (*I*_*CaL*_), rapid delayed rectifier potassium (*I*_*Kr*_), and inwardly rectifier potassium (*I*_*K*1_) currents have been identified as the main drivers of inter-individual differences in the magnitude of low-frequency APD oscillations (Sampedro-Puente et al., 2019).

Importantly, our results have provided evidence on significant effects of long-duration microgravity simulation on cardiac electrical activity. In line with previously published studies, this work has confirmed that microgravity markedly alters ventricular repolarization (D'Aunno et al., 2003; Grenon et al., 2005; Sakowski et al., 2011; Bolea et al., 2012, 2013; Caiani et al., 2013), with those alterations being more manifested when evaluated in response to sympathetic stimulation. This study adds one more T-wave characteristic to the list of ECG repolarization properties proved to be modulated by microgravity. The quantified PRD index represents a form of temporal variability in ventricular repolarization, specifically focused on oscillations of frequencies below 0.1 Hz. Although other measures of ECG temporal variability have been investigated during or immediately after simulated microgravity exposure (Sakowski et al., 2011; Bolea et al., 2013), PRD can provide a more robust characterization of repolarization instability by encompassing global T-wave vector information. Also, the PRD index, by accounting for frequencies below 0.1 Hz, has been proven to be related to sympathetic modulation of ventricular repolarization (Rizas et al., 2016). On the basis that augmented sympathetic activity is associated with adverse outcomes in different patient populations (Verrier and Antzelevitch, 2004), the evaluated PRD index is of great interest for risk prediction. The enhancement of spatial and/or temporal ventricular heterogeneities observed in this and other studies in relation to long-term exposure to microgravity conditions suggest that microgravity could accentuate repolarization instability and thus increase ventricular arrhythmic risk, especially immediately upon gravity restoration. In particular, this study has shown that PRD quantified following 60-day HDBR is highly elevated, up to 50% at rest and up to 100% in response to TTT, with respect to PRE-HDBR values. The extent of change in PRD values measured immediately after 60-day HDBR could be associated with high arrhythmic risk taking as a reference previous studies on risk assessment in post-myocardial infarction patients, where those extents of change were found in patients who died vs. those who survived during follow-up (Rizas et al., 2014, 2017). This is in line with other studies that have reported on subjects presenting long-term microgravity-induced changes in ECG repolarization of an extent similar to those associated with more than 3-fold increased hazard ratio for sudden cardiac death in general populations (Sakowski et al., 2011).

Additionally, this study has assessed two countermeasures in their ability to counteract microgravity-induced effects on ventricular repolarization. The first applied countermeasure, based on an exercise training protocol, although markedly attenuated microgravity effects as measured by changes in the PRD index, it was not able to completely reverse them. These results on partial effectiveness of exercise-based countermeasures are in line with the findings reported in Kramer et al. (2017), Maggioni et al. (2018), and Caiani et al. (2018), which investigated the same jump-based countermeasure to reverse musculoskeletal and cardiovascular deconditioning. In other studies, exercise-based countermeasures have shown to be very effective in preserving bone and muscular conditions (McRae et al., 2012; Kramer et al., 2017; Maggioni et al., 2018).

The second tested countermeasure, a nutritional supplementation composed of an anti-oxidant and anti-inflammatory dietary mix, has been shown to be far from being effective in reducing microgravity-induced effects on ventricular repolarization. This is in agreement with other studies pointing out to lack of effectiveness of this countermeasure in counteracting microgravity exposure effects on bone turnover (Austermann et al., 2019). Importantly, the intake of omega-3 fatty acids, which are components of the dietary mix, and their possible protection of cardiovascular health should additionally be viewed in relation to the potentially increased risk for ventricular arrhythmias. Such a relation is, nevertheless, controversial, with some studies suggesting that they have detrimental arrhythmogenic effects, whereas other postulate minimal effects or highly anti-arrhythmic potential (Albert, 2012; von Schacky, 2012; Coronel, 2017; Tribulova et al., 2017). Although one reason to include this type of acid in a dietary support was its protective effects on bones (Zwart et al., 2010), the findings of the present study point out that the dietary mix could not reduce adverse cardiac effects of microgravity simulation. Further studies including larger number of subjects are needed to confirm or refute these findings. Also, it is relevant to note that, when evaluating the effects of the tested countermeasures, the CTRL subgroups of the JUMP and NUTR studies did not share the same ventricular repolarization characteristics as evaluated by PRD, despite the subjects of both studies having similar physical conditions. Specifically, subjects in the CTRL subgroup of the JUMP study presented higher values of PRD, both at PRE-HDBR and POST-HDBR. Because of that reason, our results on countermeasure effects were assessed in relative terms. Nevertheless, the inclusion of a larger number of subjects would definitely allow more robust analysis of absolute and relative microgravity-induced changes. Additionally, future studies could test other types of nutritional supplements to improve the ability to counteract deleterious effects associated with long-term microgravity exposure (Cena et al., 2003). Based on the results of this study and the concordance with the outcomes of other studies, a modified jump training or a combination of exercise- and nutrition-based countermeasure (Schneider et al., 2009; Konda et al., 2019; Kramer et al., 2019), possibly including other components like pharmacological agents or artificial gravity (Evans et al., 2018), would be suggested to compensate for adverse microgravity-induced effects on ventricular repolarization.

## 5. Conclusions

The effects of long-duration microgravity on ventricular repolarization have been assessed by evaluation of the PRD index, a marker of low-frequency repolarization oscillations whose increase is related to high risk for ventricular arrhythmias and sudden cardiac death. Two methods have been developed for robust quantification of PRD, which have shown to present very good agreement. Long-term microgravity exposure has been proven to markedly elevate PRD, particularly when evaluated in response to enhanced sympathetic activity induced by a tilt table test. A countermeasure based on exercise training has been shown to partially counteract microgravity-induced changes in ventricular repolarization as assessed immediately upon gravity restoration.

## Data Availability Statement

The datasets analyzed in this article are not publicly available. Requests to access the datasets should be directed to European Space Agency.

## Ethics Statement

The studies involving human participants were reviewed and approved by Institute of Aerospace Medicine—German Aerospace Center-DLR and by Institute of Space Medicine and Physiology-MEDES. The patients/participants provided their written informed consent to participate in this study.

## Author Contributions

EP and JM devised the project, the main conceptual ideas and proof outline, and were responsible for overseeing the research and providing critical insight and recommendations regarding the focus, structure, and content of the paper. SP performed computational simulations and analyzed the data results. EC was responsible for the definition of the bed rest data acquisition protocols and contributed with technical details and analysis support. FL contributed by managing on-site data acquisition in both campaigns. All authors participated in writing and proofreading throughout the publication process.

### Conflict of Interest

The authors declare that the research was conducted in the absence of any commercial or financial relationships that could be construed as a potential conflict of interest.
