# Supplementary material for: Long-Term Microgravity Exposure Increases ECG Repolarization Instability Manifested by Low-Frequency Oscillations of T-Wave Vector
Source: Front Physiol. 2019 Dec 17;10:1510. doi: 10.3389/fphys.2019.01510 (PMC6928004; doi:10.3389/fphys.2019.01510)
Supplement: Supplementary file 1 [file Data_Sheet_1.PDF]

Supplementary Material:

Long-term microgravity exposure increases ECG repolarization instability manifested by low-frequency oscillations of T-wave vector

1 SUPPLEMENTARY TABLES AND FIGURES

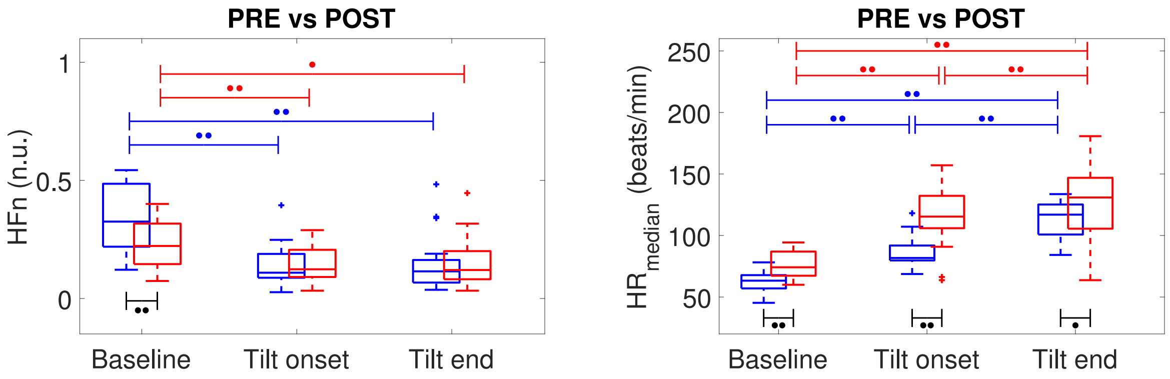

Figure S1. Boxplots of HFn and HR_{median} for the CTRL subgroups of DLR and MEDES campaigns at PRE-HDBR (in blue) and POST-HDBR (in red), evaluated at baseline and at the beginning and end of the tilt phase. $^{\bullet\bullet}$p<0.01, $^{\bullet}$p<0.05 (Wilcoxon signed-rank test)

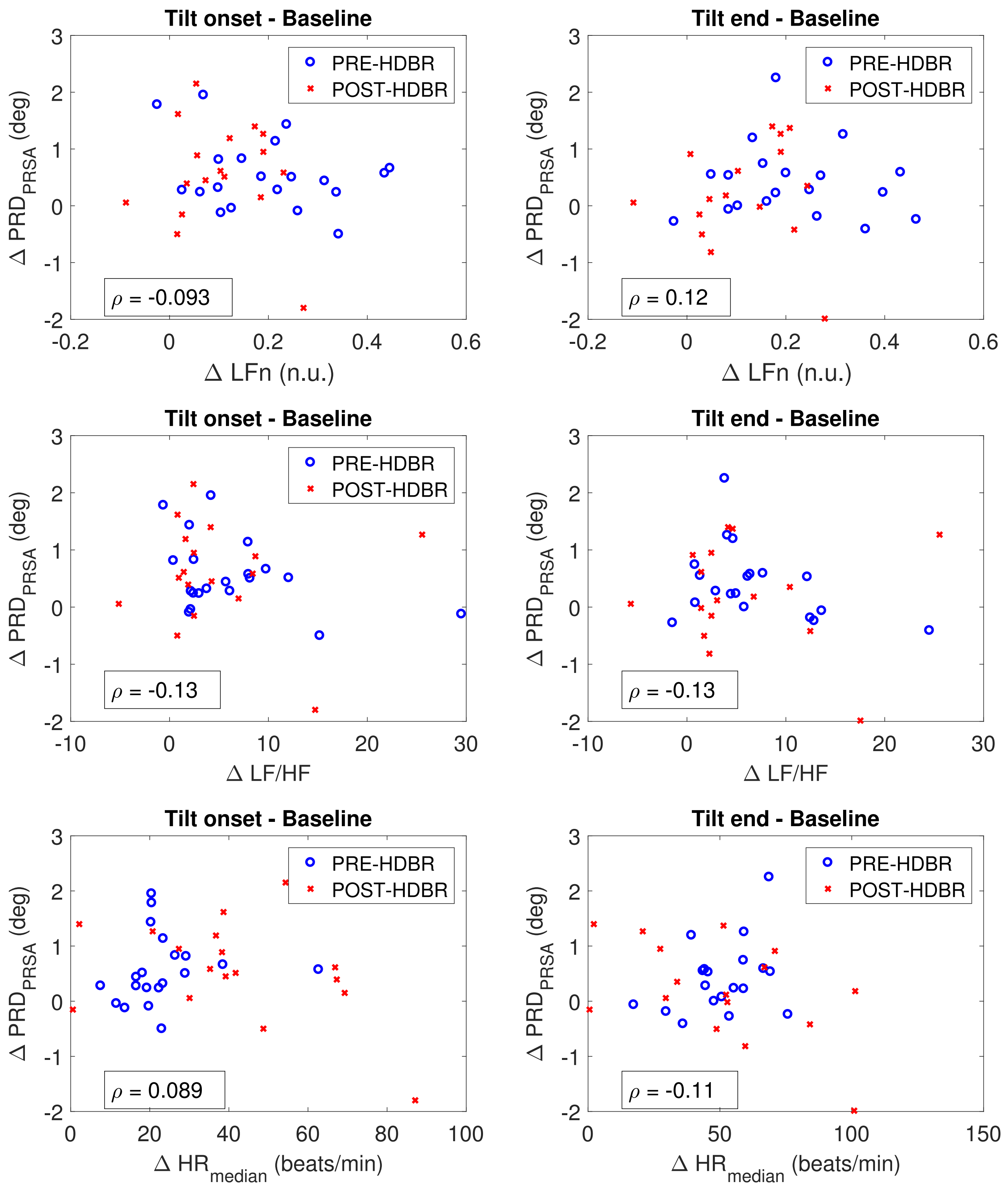

Figure S2. Scatterplots of ΔPRD_{PRSA} and change in HR or HRV indices measured as the difference between the values at the beginning of the tilt phase with respect to baseline values. Values corresponding to PRE-HDBR are shown in blue circles and those corresponding to POST-HDBR are shown in red crosses. Spearman's correlation coefficient ρ is shown for each tested association.
